# Adolescent Positivity and Future Orientation, Parental Psychological Control, and Young Adult Internalising Behaviours during COVID-19 in Nine Countries

**DOI:** 10.3390/socsci11020075

**Published:** 2022-02-14

**Authors:** Ann T. Skinner, Leyla Çiftçi, Sierra Jones, Eva Klotz, Tamara Ondrušková, Jennifer E. Lansford, Liane Peña Alampay, Suha M. Al-Hassan, Dario Bacchini, Marc H. Bornstein, Lei Chang, Kirby Deater-Deckard, Laura Di Giunta, Kenneth A. Dodge, Sevtap Gurdal, Qin Liu, Qian Long, Paul Oburu, Concetta Pastorelli, Emma Sorbring, Sombat Tapanya, Laurence Steinberg, Liliana Maria Uribe Tirado, Saengduean Yotanyamaneewong

**Affiliations:** 1 Center for Child and Family Policy, Duke University, Durham, NC 27708, USA; 2 Institute for Psychotherapy, Medical School Berlin, 14197 Berlin, Germany; 3 Department of Psychology, Duke University, Durham, NC 27708, USA; 4 Clinical Psychology, Utrecht University, 3584 CS Utrecht, The Netherlands; 5 Division of Psychiatry, University College London, London W1T 7NF, UK; 6 Department of Psychology, Ateneo de Manila University, Quezon City 1008, Philippines; 7 Department of Special Education, Hashemite University, Zarqa 13110, Jordan; 8 Department of Humanistic Studies, University of Naples “Federico II”, 80127 Naples, Italy; 9 Eunice Kennedy Shriver, National Institute of Child Health and Human Development, Bethesda, MD 20810, USA; 10 UNICEF, New York, NY 10001, USA; 11 Institute for Fiscal Studies, London WC2R 2PP, UK; 12 Department of Psychology, University of Macau, Macau 999078, China; 13 Department of Psychological and Brain Sciences, University of Massachusetts, Amherst, MA 01002, USA; 14 Department of Psychology, Università di Roma La Sapienza, 00017 Rome, Italy; 15 Centre for Child and Youth Studies, University West, 46131 Trollhättan, Sweden; 16 Maternal and Child Health, School of Public Health and Management, Chongqing Medical University, Chongqing 400016, China; 17 Global Health Research Center, Duke Kunshan University, Kunshan 215300, China; 18 Department of Psychology, Maseno University, Maseno 879-6112, Kenya; 19 Peace Culture Foundation, Chiang Mai 50000, Thailand; 20 Department of Psychology, Temple University, Philadelphia, PA 19019, USA; 21 Department of Psychology, King Abdulaziz University, Jeddah 22230, Saudi Arabia; 22 Department of Psychology, Universidad de San Buenaventura, Medellín 050001, Colombia; 23 Department of Psychology, Chiang Mai University, Chiang Mai 50000, Thailand

**Keywords:** parenting, COVID-19, 21st century, adolescence, internalising

## Abstract

The COVID-19 pandemic disrupted many young adults’ lives educationally, economically, and personally. This study investigated associations between COVID-19-related disruption and perception of increases in internalising symptoms among young adults and whether these associations were moderated by earlier measures of adolescent positivity and future orientation and parental psychological control. Participants included 1329 adolescents at Time 1, and 810 of those participants as young adults (*M* age = 20, 50.4% female) at Time 2 from 9 countries (China, Colombia, Italy, Jordan, Kenya, the Philippines, Sweden, Thailand, and the United States). Drawing from a larger longitudinal study of adolescent risk taking and young adult competence, this study controlled for earlier levels of internalising symptoms during adolescence in examining these associations. Higher levels of adolescent positivity and future orientation as well as parent psychological control during late adolescence helped protect young adults from sharper perceived increases in anxiety and depression during the first nine months of widespread pandemic lockdowns in all nine countries. Findings are discussed in terms of how families in the 21st century can foster greater resilience during and after adolescence when faced with community-wide stressors, and the results provide new information about how psychological control may play a protective role during times of significant community-wide threats to personal health and welfare.

## 1. Introduction

The COVID-19 pandemic disrupted the lives of families around the world, presenting specific challenges to young adults. Following the widespread and rapid outbreak of the SARS-CoV-2 infection, young adults were confronted with swift and severe changes in daily routines. For many young adults, the pandemic meant pauses in education or cessation of in-person interactions during learning, changes in residence (e.g., moving back home with parents) and reduced access to health care, leisure activities, mental health services, as well as other social supports. The role of thinking about the future in a positive way is vital to adaptive adjustment in the transition from adolescence to young adulthood and greater independence (Steinberg et al. 2009). Because the pandemic disrupted the important transition for many late adolescents into young adulthood, understanding adolescents’ thoughts about the future may be a window to understanding how young adults will adapt to major negative life events, and may inform parenting practices that may mitigate long-term negative psychological effects of future community-wide stressors or public health crises.

Parenting research and practice in the 20th century progressed from a focus on physical health, which dominated the 19th and early part of the 20th centuries, to emphases that included attachment and family relationships by the end of the 1940s (see Kaplan and Owens 2004 for overview). As decades passed, research stressed the importance of child temperament, parenting styles, cognition—including a focus on parenting involvement in education—agency, self-regulation, and prosocial behaviour. The COVID-19 pandemic has already had a profound influence on the direction of parenting and child development research. A large body of literature has emerged focusing on parent–child relationships, family functioning, and regional differences among how families are adjusting during the pandemic (Weeland et al. 2021). However, the pandemic is not the only influence on parenting and family functioning; economic uncertainty, climate change, increased globalisation, and modernisation of and access to technology all compete as stressors as children progress through adolescence into adulthood. Disruptions in what were once assumed to be typical developmental pathways may become even more difficult to predict. In addition to concerns about how young adults now view their future, the unpredictability of the current pandemic and the influences listed above lead us to re-examine aspects of the parent–child relationship that may influence adjustment as adolescents enter adulthood in an uncertain world.

## 2. COVID-19 and Mental Health

Evidence from the first months of the pandemic indicates increased rates of internalising symptoms among people from various countries and age groups (Islam et al. 2021; Ravens-Sieberer et al. 2020). A longitudinal study of internalising symptoms in adults showed increased rates of anxiety and depression during the COVID-19 pandemic lockdown; these increases were observed continually throughout the course of the pandemic (Andersen et al. 2021).

The pandemic also affected whole families and communities. At the family level, the pandemic caused reorganisation of daily routines, cuts in external support by other family members and social support systems, fear of losing family members and in the case of deaths, normal bereavement and grief processes were disrupted if not completely absent (Fegert et al. 2020). As such, stressors during the pandemic increased, whereas the opportunities to regulate stress through outside or social activities fell apart or were completely lost.

A meta-analysis of 29 studies assessing more than 80,000 youth globally showed a higher prevalence of clinically significant symptoms of depression and anxiety after the onset of the COVID-19 pandemic compared to prior estimates (Racine et al. 2021). For example, the global estimates of depression (12.9%) and anxiety (11.6%) prior to the pandemic were much lower compared to during the pandemic (depression, 25.2%; anxiety, 20.5%). Although most of the studies in this analysis were specific to China, some included participants from North America, Europe, and other Asian countries. Similar increases have been observed in other countries. For instance, a study from Italy indicated high rates of depressive (47.5%) and anxious (14.1%) symptoms among 326 adolescents aged 14–19 years (Pisano et al. 2021). These rates are considerably higher than evidence from previous Italian epidemiological studies, which indicate the prevalence of internalising symptoms to be much lower before the pandemic (e.g., Frigerio et al. 2009; Gritti et al. 2014).

Several studies indicate that young people are at higher risk of having increased internalising symptoms compared to adults and older age groups. For example, Smith et al. (2020) identified several risk factors associated with poor mental health during the pandemic, namely younger age, female gender, lower annual income, current smoking, and the presence of physical multimorbidity. Further, a cross-sectional study in UAE found that the highest levels of generalised anxiety disorder (GAD) were among young people (71%) and females (51.7%) (Saddik et al. 2021). Higher levels of anxiety symptoms were found among those who worried about their parents or children contracting COVID-19 and transmitting COVID-19 to someone else if they contracted the virus. Other research highlights adjustment difficulties related to COVID-19 specific to young adults. In a study of 450 college students in the mid-Atlantic United States, an area hard-hit by the pandemic during 2020, results revealed that young adults reported increases in their inability to focus, increased anxiety and depression, and excess time spent searching for information about COVID-19 (Kecojevic et al. 2020). A study with college students in China noted that family economic difficulties related to COVID-19 were related to parent–child relationship difficulties, which in turn were related to increases in self-reported anxiety and depression (Cui and Hong 2021).

## 3. COVID-19, Positivity, and Future Orientation

The COVID-19 pandemic challenges future thinking and behaviour in part because conditions during the pandemic present young adults with a high degree of uncertainty and challenge their optimism and future outlook. Positivity is defined as the degree to which an individual has an affirming regard for their own abilities and qualities, confidence that others will support them in their goals, and a general outlook on life that is hopeful and optimistic (Caprara et al. 2012). Future orientation has many additional components, including cognitive (How much do I think about the future?), attitudinal (How much am I willing to give up now to wait for a better outcome later?), and motivational goals (How much time do I spend planning to achieve long-term goals?) (Steinberg et al. 2009). Further, the development of future orientation is influenced by several factors, including adolescents’ social and family environment (Seginer 2009). Future-oriented thinking and behaviour are associated with less maladjustment during childhood and adolescence (Hamilton et al. 2015; Holman and Silver 2005). In the sections that follow, we review the literature on positivity and future orientation and their application to the COVID-19 pandemic.

### 3.1. Positivity

Positivity is generally understood to be protective against psychosocial maladaptation, especially when faced with stressful events (Caprara et al. 2019; Milioni et al. 2016). It is a relatively stable trait across developmental periods (Alessandri et al. 2012), and is linked to better physical health (Caprara et al. 2017). In contrast to future orientation, which identifies specific behaviours, positivity is a basic attitude needed to face major challenges (Caprara et al. 2019) and includes both self-esteem and optimism.

The COVID-19 pandemic presents young adults with ongoing and serious threats to predictability and safety. In the face of uncertainty, adaptive coping skills are important predictors of positive adjustment, and positive reappraisal of negative experiences can aid this coping (Shing et al. 2016). Positivity, however, is not simply the absence of negative thought or negative emotion, and positivity does not signal the absence of depression, much in the same way well-being is not defined only by the absence of illness (World Health Organization 2004). Rather, the presence of positive emotions that outweigh negative ones, optimistic thoughts, and general life satisfaction can be protective against depression, as shown in both longitudinal and daily diary studies (Alessandri et al. 2014; Caprara et al. 2019; Fredrickson et al. 2003). For example, following the terrorist attacks in the United States on 9/11, the difference between resilient and non-resilient individuals was that resiliency was characterised by more positive emotions than negative ones, not just the complete absence of any negative thoughts (Fredrickson et al. 2003). In a study of middle school students over three years, optimism about the future was associated with decreased internalising symptoms (Smokowski et al. 2017). In 475 undergraduate students in Turkey during the COVID-19 pandemic, optimism mediated the relation between pandemic stress and depressive symptoms (Arslan and Yıldırım 2021). Not all associations between positivity and mental health are encouraging, however. Research examining positive future fantasies about daily life and academic achievement, among other things, concludes that when such fantasies lower effort and success, they can have an exacerbating impact on depression (Oettingen et al. 2016). Thus, it appears that positivity is linked with lower internalising symptoms overall, but the effect is more nuanced if the optimism creates unrealistic visions of success that lower effort and undermine goal achievement.

### 3.2. Future Orientation

Future orientation has long been studied in both developmental and cultural contexts (see Mönks 1968; Seginer 1986, among others). Development throughout late adolescence and young adulthood includes several future-oriented decisions and milestones related to family life, education, and work (Nurmi 1991). The COVID-19 pandemic disrupted all aspects of life related to typical developmental milestones and presented challenges to thinking about life beyond the immediate present; young adulthood is thus an ideal time to examine the impact on well-being during the pandemic via potential disruptions in typical developmental processes (Steinberg et al. 2009). A sense of hopelessness and feelings of lack of control can set in during community-wide stressors (So et al. 2018) and lead young adults to have diminished beliefs about a positive future.

However, future-oriented thinking can be a protective factor in the face of difficulties. For example, with no clear timeline and widespread uncertainty about pandemic-related restrictions and infection spread, young adults may need to reframe their thoughts about the challenges: Present restrictions can be better accepted if they are seen as a means of securing a more positive future outcome (Lalot et al. 2021). People who think a lot about their future may be better prepared when obstacles get in their way and may be less vulnerable to depression following high levels of stress (Johnson et al. 2014). Empirical findings support this hypothesis. Cross-sectionally, higher future orientation moderated the association between daily stress and symptoms of depression in college students (Zheng et al. 2019). Over time, future-oriented thinking and feelings of agency in both youth-at-risk (Stoddard et al. 2011) and the general population during COVID-19 (Lalot et al. 2021) were associated with better well-being and fewer externalising behaviours. One explanation may be that anticipating future consequences may allow individuals to focus on goals and thus avoid behaviours that reduce the likelihood of attaining them (Zheng et al. 2019). Future-oriented thinking may also be helpful during the pandemic because it allows individuals to shift their focus from present stress to anticipation of future happiness, and thus improves emotion regulation (MacLeod and Conway 2005). Much of the literature on the impact of future orientation during periods of stress is cross-sectional, however, and limited in cross-national range. Further, little is known about the long-term associations between future orientation and coping with major stressors later in life.

## 4. Parental Psychological Control

Parental psychological control (e.g., intrusion, love withdrawal, and guilt induction) plays a key role during the adolescent years on the child’s psychological and emotional development (Barber 1996; Barber et al. 1994; Smetana 2017). Parental control can be distinguished into behavioural control, which refers to monitoring and limit setting, and psychological control, which refers to the degree of emotional autonomy that parents grant to the child (Gray and Steinberg 1999). In this paper, we focus on psychological control, which includes any emotionally and psychologically manipulative strategies or behaviours of parents that disregard the child’s autonomy and disrupt the child’s volitional functioning (Barber and Xia 2013; Soenens and Vansteenkiste 2010). Such parenting techniques have a detrimental effect on children’s development, future adjustment, and internalising and externalising symptoms (Arredondo et al. 2006; Chao and Aque 2009; Mandara and Pikes 2008; Silk et al. 2003). For instance, Rogers et al. (2003) found that disrupted adjustment and elevated levels of internalising are mostly reported in situations when both parents are high in psychological control. Moreover, it seems that parental psychological control is directly connected to development of depressive symptoms rather than children’s resilience. Although parental psychological control has been widely studied among children and adolescents, it is important to understand the impact of psychological control during later developmental stages, such as emerging adulthood. Parental psychological control continues to be relevant during young adulthood because it can be expressed from a distance and affect the individual’s self-sufficiency, emotional regulation, life satisfaction, and endorsement of adulthood status (Faherty et al. 2020; Manzeske and Stright 2009; Sholomskas and Axelrod 1986).

The COVID-19 pandemic exposed families and young adults to additional stressors and adversity, due to increased family demands, heightened levels of uncertainty, and drastic change of family routines that all impede the families’ adaptive capacity (Masten and Motti-Stefanidi 2020). Indeed, emerging adults, aged 18 to 30 years, who reported higher parental psychological control, showed negative reactivity to the pandemic (Ma and Wang 2021). Despite the current cross-sectional evidence, it is not yet clear whether the effects of parental psychological control last over time. Thus, in the current study, we investigate whether higher levels of psychological control during adolescence interfere later in life with a young adult’s adjustment to the COVID-19 pandemic.

## 5. COVID-19 in Context

Together, the literature about positivity, future orientation, and coping during community-wide stressors supports the idea that future-oriented thinking and behaviour can be protective against symptoms of anxiety and depression. The literature is less clear about how parental psychological control may impact young adult adjustment. Although the extant literature on control is clear that higher levels of parental psychological control are most often associated with maladjustment in youth, the impact of a community-wide stressor remains to be examined across a diverse sample. To examine these findings in relation to the COVID-19 pandemic across a range of countries, we utilised data from a cross-national study of parenting, adolescent risk taking, and young adult competence that included pre-pandemic data about positivity, future orientation, parental psychological control, and prior levels of adolescent internalising behaviours. The countries included in this study—China, Colombia, Italy, Jordan, Kenya, the Philippines, Sweden, Thailand, and the United States—have had varied experiences with infection rates, government response in the form of restrictions, school closures, community lockdowns, and vaccine distribution since the onset of the pandemic. We recognise that the pandemic situation in each country continues to evolve as new variants, access to vaccines, and other parameters contribute to change over time. The information below reflects the situation in each region earlier in the pandemic closer to the time of data collection.

Southeast Asian countries that were able to limit widespread infection in 2020 were in the third quarter of 2021 dealing with overburdened health-care systems, a lack of hospital beds, equipment, and oxygen (Regan 2021). Many countries also reinstated rigorous lockdowns to slow the spread of the Delta variant of the virus. Thailand and the Philippines were able to relax tight lockdown requirements after keeping infection counts low in 2020, but by August 2021 the Philippines had the highest number of COVID-19 cases in Southeast Asia. In Eastern Asia, China tried to avoid full city lockdowns, but reimposed considerably tighter social distancing measures and COVID guidelines as the Delta variant spread throughout more than half of its provinces (Gan 2021).

In Europe, Italy was the first country to experience widespread infection and death after the initial coronavirus outbreak in China. Italy imposed containment procedures quickly after the initial 2020 outbreak that helped mitigate the spread of COVID-19 (McCann et al. 2020). Sweden closed secondary—but not primary—schools and universities, and businesses overall did not experience widespread initial closures (Goodman 2020). By August 2021, both countries have eased pandemic restrictions greatly despite their differing initial responses to the pandemic. Sweden continued to ease pandemic restrictions in August 2021, which has included continually lifting mask recommendations (Fulton 2021). To date, there has been no community-wide mask mandate. Similarly, Italy has continued to ease restrictions on mandates and instead of forcing shutdowns is implementing new measures to try to curb the spread of infection. These measures include a digital COVID-19 vaccination certificate that will be mandatory in Italy’s restaurants and other public spaces (Bubola 2021).

Countries in the Middle East and Africa that were relatively successful in containing the spread of COVID-19 by responding strictly and promptly are now repeating their efforts as cases are on the rise again. In comparison to neighbouring countries, Jordan initially mostly evaded the COVID-19 pandemic. The Jordanian government enforced strict restrictions, including mandatory curfews, suspension of international travel, and the closure of schools and businesses (Santucci 2020). As of August 2021, Jordan’s COVID-19 infection and death rates were increasing (Best 2021). Recommendations to close schools and workplaces and stay-at home orders were on the rise (Reuters 2021). In response to the Delta variant, the Western region of Kenya was under lockdown by August of 2021. The Kenyan government reacted quickly in the previous waves of COVID and continued to do so while also providing consistent messaging and updates to residents (Mohiddin et al. 2020). As of August 2021, public gatherings and in-person meetings were suspended countrywide, and Kenya extended its national curfew (Al Jazeera 2021).

The Americas, specifically Colombia and the United States, have been feeling the devastating effects of COVID-19 and are still battling the disease outbreak. The Colombian government imposed a strict national quarantine and lockdown in response to the COVID-19 crisis in 2020. Businesses ceased operations, a public mask mandate was issued, and the state of emergency was extended (Rueda 2021). In August 2021, Colombia was dealing with the longest COVID-19 wave to date, with intensive care units (ICUs) at 95% capacity. Despite this, Colombia and other South American countries began to reopen their economies, though vaccine access remained limited (Lopez-Carr 2021). During August 2021, the COVID-19 outbreak in the United States reached 100,000 new confirmed daily infections, a record set during the winter surge fuelled by the highly transmissible Delta variant and low vaccination rates in the southern U.S. states (Spencer and Kennedy 2021).

## 6. Present Study

This study utilises self-report data from adolescents and young adults and their mothers and fathers over a three-year period in a diverse group of nine countries to assess associations among COVID-19 personal disruption and perceived increases in internalising symptoms in young adulthood, and whether positivity, future-oriented thoughts and behaviour, or parental psychological control during adolescence moderate those relations. In line with prior research during experiences with community-wide stressors, including natural disasters (Bermudez et al. 2019; Hafstad et al. 2012), 9/11 (Calderoni et al. 2006; Hendricks and Bornstein 2007), and the SARS outbreak (Hawryluck et al. 2004), we hypothesised that high levels of pandemic-related disruption during young adulthood would be related to perceived increases in internalising symptoms. Because the literature identified a gap in our cross-national understanding of how positivity and future orientation are associated with internalising behaviours during the pandemic, we also examined whether positivity and future orientation during late adolescence moderated the disruption–internalising link. We predicted that, across countries, the association between pandemic-related disruption and increases in internalising behaviour during young adulthood would be moderated by prior levels of both positivity and future orientation during adolescence, even when controlling for adolescents’ prior pre-pandemic levels of internalising symptoms, such that more positivity and higher levels of future orientation during adolescence would buffer the association between pandemic-related disruption and increases in internalising symptoms. Further, we predicted that psychological control imposed by parents during adolescence could impair young adults’ response to the COVID-19 pandemic and its stressors and would moderate the relation between disruption and perceived increases in internalising such that higher levels of parental psychological control during adolescence would be related to a greater perceived increase in internalising during the pandemic for young adults.

## 7. Method

### 7.1. Participants

**Figure 1.**
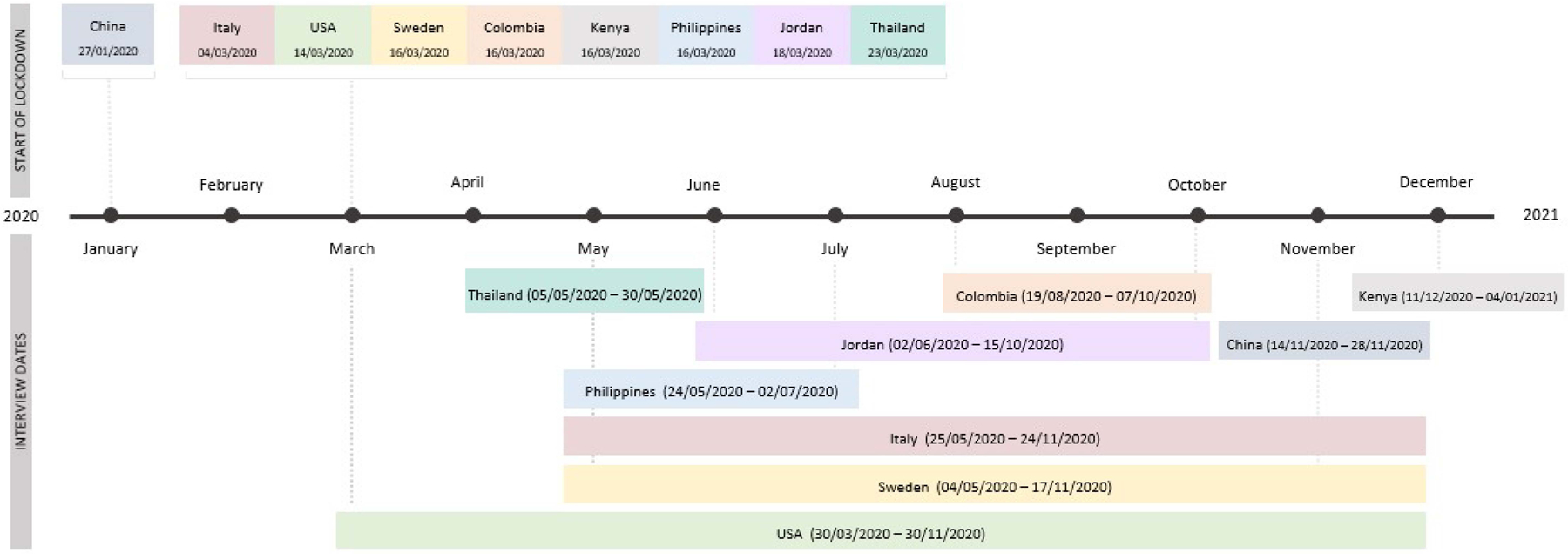
Timeline of COVID Lockdowns and Data Collection Dates.

Participants for this study were 1329 youth at Time 1 (50.4% female) and their mothers and fathers who were drawn from a larger study (see Lansford et al. 2016 for more information). Time 1 data collection occurred over approximately a 13 month period when youth were, on average, between 16.67 (*SD* = 0.96) and 17.75 (*SD* = 1.02) years old. To reduce the time burden on participants, psychological control, prior level of interalising behaviour and positivity were collected at age 16, and future orientation was collected at age 17. At Time 2, which occurred during the COVID-19 pandemic, young adults were on average 20.00 years old (*SD* = 1.16). Participants were recruited from the following sites: Chongqing, China (*n* = 114), Medellín, Colombia (*n* = 108), Rome and Naples, Italy (*n* = 213), Zarqa, Jordan (*n* = 114), Kisumu, Kenya (*n* = 100), Manila, Philippines (*n* = 120), Trollhättan/Vänersborg, Sweden (*n* = 129), Chiang Mai, Thailand (*n* = 120), and Durham, North Carolina, United States (*n* = 311). On average, parents had 13.71 years of education (*SD* = 4.18). Across countries, schools had been closed for an average of 20.41 weeks (*SD* = 12.07) due to pandemic-related lockdowns when young adults completed the COVID measure. Figure 1 shows a timeline of lockdowns and data collection dates for each country.

Due to rapid data collection endeavours and difficulties with interviewing in person during the pandemic, data were collected from 810 young adults (60.9%) at age 20 between late March 2020 and early January 2021. Young adults who participated at age 20 did not significantly differ from adolescents who participated at age 16 with regard to their prior levels of internalising behaviours, t(1040) = −1.91, *p* = 0.055, or their parents’ years of education, t(1316) = −0.436, *p* = 0.66. However, participants at age 20 were more likely to be female than participants who did not provide age 20 data, χ^2^ (1) = 16.99, *p* < 0.001.

### 7.2. Procedures

Prior to beginning interviews in all countries, ethics board review was obtained through each participating university. To ensure linguistic and construct equivalence of each measure, translators fluent in English and the target language provided forward- and backward-translation of items (Erkut 2010). When youth were adolescents, trained interviewers in each site contacted families to conduct individual interviews in person, by telephone, by mail, in writing, or via a web-based interview, according to participant preference. Informed consent was provided by parents until young people reached majority age and could provide their own consent. Adolescent and parent interviews at Time 1 lasted approximately 60–90 min. At age 20, all sites were experiencing varying restrictions due to pandemic-related lockdowns, and no in-person interviews could be conducted. Instead, using telephone, written, or web-based surveys according to participant preference, interviewers captured a brief assessment of COVID-19-related experiences with a 19-item questionnaire lasting no more than 5 min. Participants were provided small stipends in appreciation for their time.

### 7.3. Measures

#### 7.3.1. Positivity

During adolescence, youth responded to an 8-item Positivity Scale (Caprara et al. 2012). Each item was scaled from 1 = strongly disagree to 5 = strongly agree to measure how positively the adolescents felt about themselves and the future. Sample items included, “I have great faith in the future”, “I feel I have many things to be proud of”, and “I am satisfied with my life”. A mean score was derived from 8 items, with one item reverse-scored. The scale had good reliability in our sample (α = 0.84). Previous work established internal and construct validity, test–retest reliability, and measurement invariance across cultures (Caprara et al. 2012).

#### 7.3.2. Future Orientation

Adolescent future orientation was measured using 15 items from the Future Orientation Scale (see Steinberg et al. 2009 for psychometrics and full scale). To reduce socially desirable responding, the measures used formatting originally designed by Harter (1982). Questions were asked in two parts; first, adolescents chose which statement best described them, with opposing statements separated by “BUT”, i.e., “Some people like to plan things out one step at a time, BUT other people like to jump right into things without planning them out beforehand”. Next, the respondent indicated if the statement they chose was sort of true for them or really true for them. The result was a 4-point scale ranging from really true on one side of the statement to really true on the other side of the statement. The full scaled score was a mean of all 15 items covering time perspective, planning ahead, and anticipation of future consequences. Reliability in our sample was good (*α* = 0.78).

#### 7.3.3. Psychological Control

Mothers and fathers reported about the degree to which parents utilised non-coercive discipline techniques and encouraged adolescents to express autonomy. Eleven items were adapted from earlier work (Barber 1996; Dornbusch et al. 1985; Patterson and Stouthamer-Loeber 1984; Rodgers 1966; Silk et al. 2003). Mothers and fathers were asked to respond separately about the target child; items were scored from 1 = strongly disagree to 4 = strongly agree. Items included “I say that my child should give in on arguments rather than make people angry”, and “I tell my child that my ideas are correct and that he/she should not question them”. Higher scores indicated higher levels of parental psychological control and lower levels of adolescent autonomy. To reduce the number of models, and because parent–child dyadic differences were not of primary interest in this study, a mean of mother and father responses (*α* = 0.77) was created. Psychometrics of the original factor structure across multiple contexts are reported elsewhere (Steinberg et al. 1991).

#### 7.3.4. Experiences during COVID-19

At age 20, young adults completed *Experiences with COVID-19*, a measure designed to provide a quick assessment of perceived changes in psychosocial functioning, interpersonal relationships, attitudes, and behaviours related to pandemic restrictions, and other personal experiences with the pandemic; the development of this measure based on other community-wide disasters and stressors is described elsewhere (Skinner et al. 2021). Depending on the site, young adults completed this measure between late March of 2020 and early January of 2021.

COVID-19 disruption. Young adults were asked to rate their own personal experience with disruption due to the pandemic on a scale of 1 to 10, with 1 = not at all disruptive and 10 = extremely disruptive. Respondents were asked to consider changes in schooling, family, work, and other routines within a single item, to capture a generalised feeling of overall disruption. More detail about the source of the stressor (e.g., work vs. school) may be useful in other studies, but our main area of concern was overall disruption and its possible association with adjustment. Thus, brevity in survey administration time was prioritised in keeping to a single item.

Perceived increases in internalising behaviour. Young adults responded to two items assessing their perceptions about increases in their own feelings of anxiety and depression “now as compared to before the outbreak of COVID-19 in your community”. Self-reported perceptions of changes in internalising symptoms have been shown to be correlated with more objective measures of anxiety and depression (Zimmerman et al. 2004). An average score between two items (*r* = 0.53, *p* < 0.01) was created from “I feel more anxious now than I did before the outbreak” and “I feel more depressed now than I did before the outbreak”. Respondents reported on a four-point scale ranging from 1 = strongly disagree to 4 = strongly agree. Higher means indicated self-reported perceived increases in internalising behaviour at the time of interview compared to before pandemic onset. These 2 perceived changes in internalising items have been utilised by other research teams examining mental health during the pandemic (e.g., Davidson et al. 2020; Kapetanovic et al. 2021).

#### 7.3.5. Covariates

Using the Achenbach Youth Self-Report of the Child Behaviour Checklist (Achenbach 1991), we controlled for prior levels of internalising behaviour measured at age 16 using the 29 internalising items. Additionally, we controlled for the number of weeks that had elapsed since the onset of the pandemic in each site, which ranged from 1 to 40 weeks across all respondents, as this may impact perceptions of disruption in each location. We also controlled for highest level of education reached by either parent (as a proxy for SES) and adolescent gender.

### 7.4. Statistical Analysis

**Figure 2.**
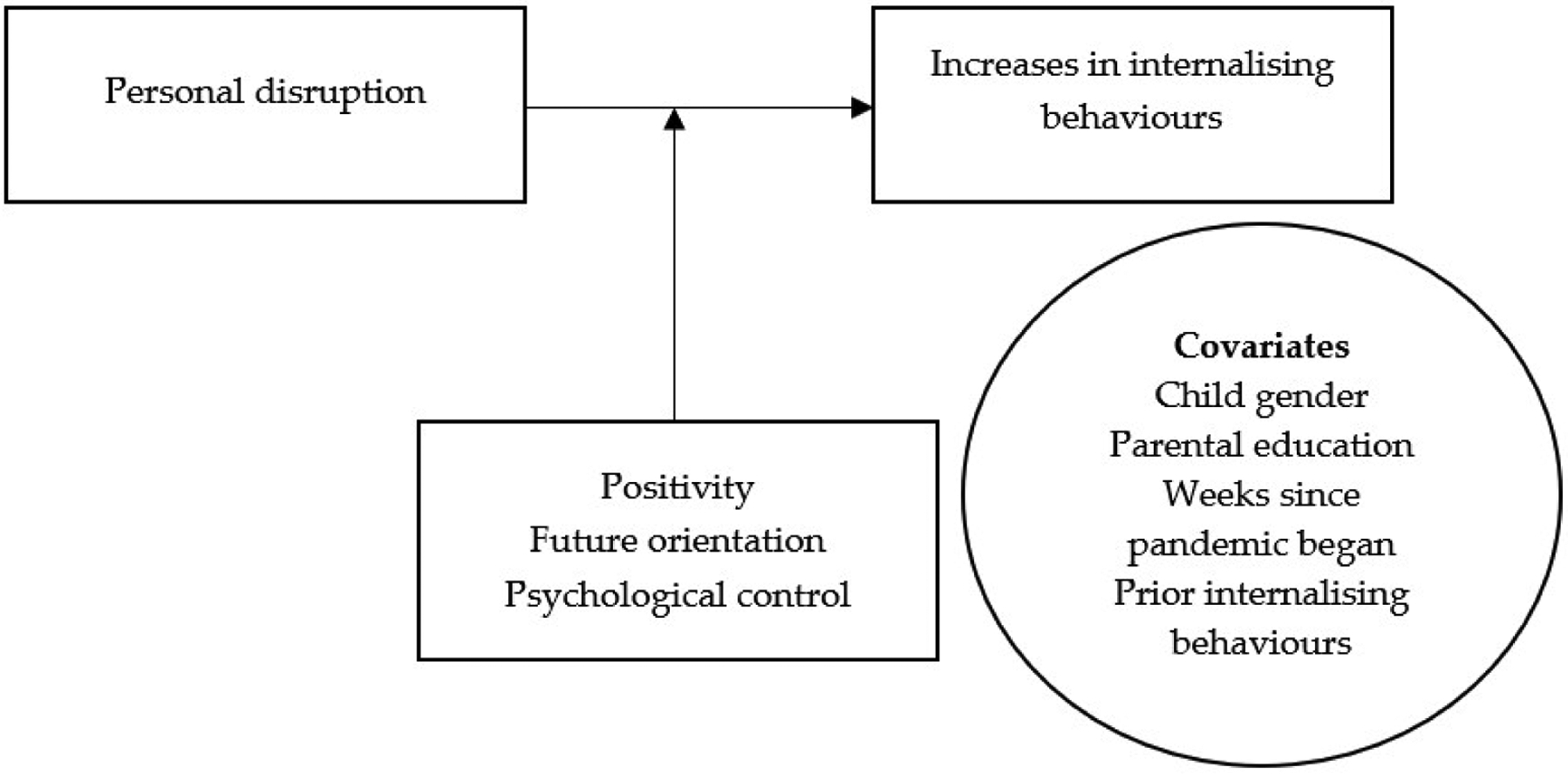
Statistical Model.

All preliminary analyses, including descriptive statistics and Pearson’s correlations, were conducted in SPSS Version 23.0. M*Plus* Version 8.4 (Muthén and Muthén 2017) was used for all further analyses. Missing data were handled via full information maximum likelihood estimation because it uses all available information to estimate each model parameter, thus making it an appropriate choice if the data are either MAR or MCAR (Kelloway 2014; Schafer and Graham 2002). All variables were standardised before further analyses. Figure 2 displays the statistical model. A multigroup structural equation model, with countries presenting nine different groups, was conducted to examine our research questions. Because the groups are not nationally representative, we do not have specific hypotheses about how each country may differ in response to the pandemic, but we are able to identify those countries whose slopes and intercepts differed from the others. The model included the association between pandemic-related disruption and changes in youth internalising behaviours as well as all control variables (parents’ level of education, weeks since the pandemic began, adolescent gender, and prior levels of internalising behaviours), and the moderators positivity, future orientation, and psychological control. To test the interaction between disruption and positivity, future orientation, or psychological control, three subsequent analytical models were run that included the respective interaction term and the other potential moderators as predictors. The model constrained the path coefficients to be equal across countries but allowed the intercepts, covariances, and residual variances to vary by country. Sample sizes, means, and correlations of all study variables are presented in Table 1.

Model fit was assessed with four fit indices: (1) the comparative fit index (CFI), (2) the Tucker–Lewis index (TLI), (3) the root mean square error of approximation (RMSEA), and (4) the standardised root mean square residual (SRMR). These multiple fit indices were used because they examine different types of model fit and, when used together, provide a more reliable assessment (Brown 2006). Recommended guidelines for each model fit index were used: CFI/TLI > 0.95, RMSEA < 0.06, and SRMR < 0.08 represented good fit (Kline 2005). When the model did not fit the data well, country-specific coefficients were released based on modification indices and theoretical meaningfulness until good model fit was achieved. A *p*-value ≤ 0.05 was used to make inferences about statistical significance. To interpret effect sizes of all estimates, guidelines by Cohen (1992) were used. Values of 0.10 are considered a small effect, values of 0.30 are considered a medium effect, and values of 0.50 or more are considered a large effect. We created the interaction plots by graphing 1 *SD* above the mean of the moderator and 1 *SD* below the mean.

## 8. Results

### 8.1. Main and Moderation Effects

On average, positivity and future orientation were high across all participants. Internalising behaviours during adolescence were strongly negatively correlated with positivity during adolescence, and moderately correlated with perceived increases in internalising behaviours during young adulthood. Positivity and future orientation during adolescence showed a small correlation. Both positivity and future orientation showed small correlations with personal disruption during young adulthood. Parental psychological control and positivity were weakly correlated.

Model fit statistics suggested good fit of all models (Table 2). Parameter estimates of both models can be found in Table 2.

#### 8.1.1. Perceived Increases in Internalising Behaviours

Significant positive associations were found between the experience of disruption due to COVID-19 and perceived increases in internalising behaviours for adolescents across all countries. Higher levels of experienced disruption due to COVID-19 were thus related to higher perceived increases in internalising behaviours in young adults (*b* = 0.27; *SE* = 0.03, *p* < 0.01).

#### 8.1.2. Positivity

Positivity during adolescence was significantly related to perceived increases in internalising behaviours in young adults across all countries except Italy (*b* = 0.12; *SE* = 0.046, *p* < 0.05). For adolescents in Italy, more positive views about life were negatively associated with perceived increases in internalising behaviours (*b* = −0.17, *SE* = 0.07, *p* < 0.05).

**Figure 3.**
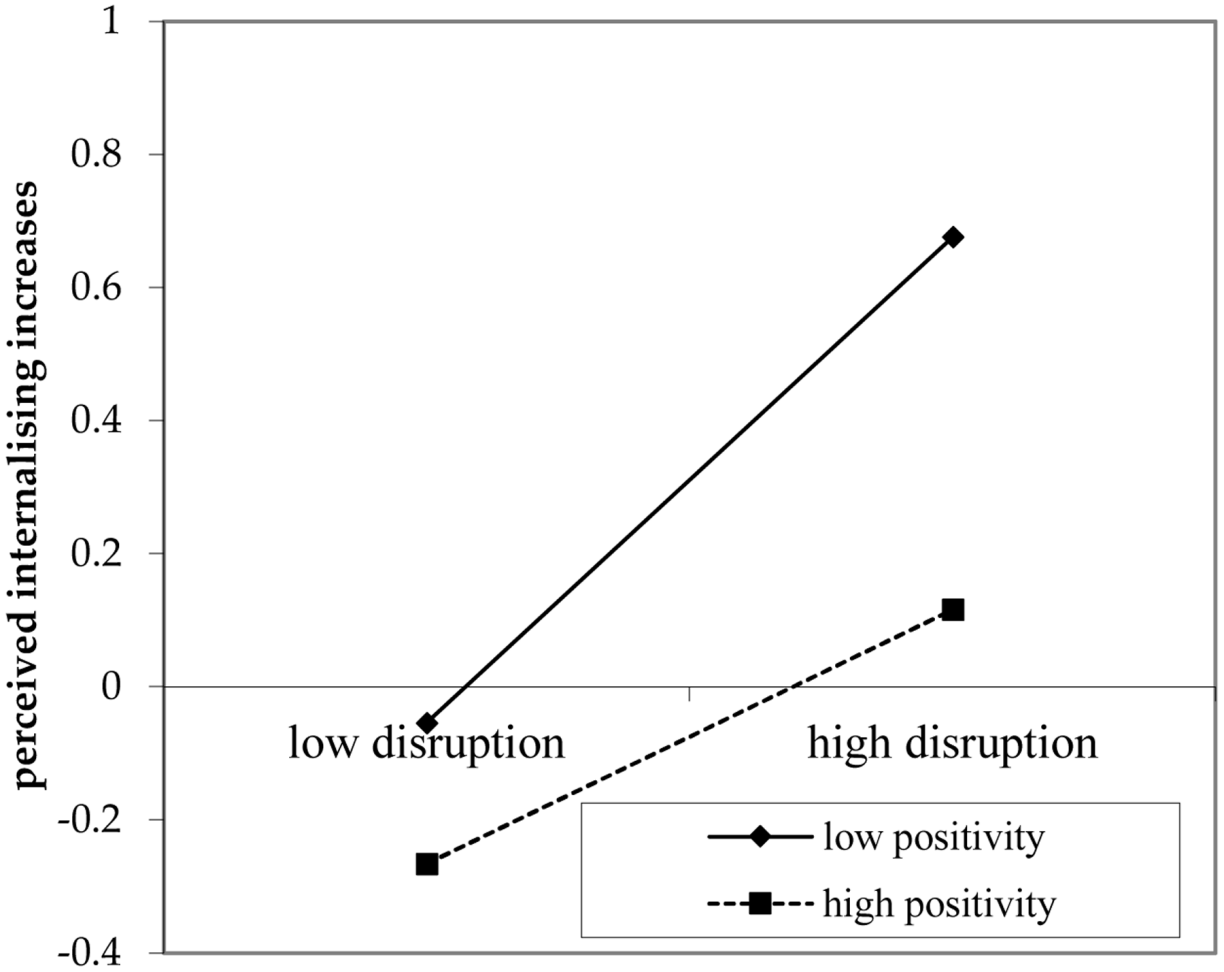
Moderation by Positivity.

There was consistent evidence across countries that the relation between experienced disruption due to COVID-19 and perceived increases in internalising behaviours in young adults was moderated by adolescents’ positivity (see Figure 3). Higher levels of personal disruption due to the pandemic were associated with greater perceived increases in internalising behaviours among adolescents who had less positive views about life (*b* = −0.09, *SE* = 0.03, *p* < 0.05). This association had a small effect size (Cohen 1992). High levels of positivity during late adolescence may thus have a protective role in the association between disruption due to COVID-19 and perceived increases in internalising behaviours in young adults.

#### 8.1.3. Future Orientation

**Figure 4.**
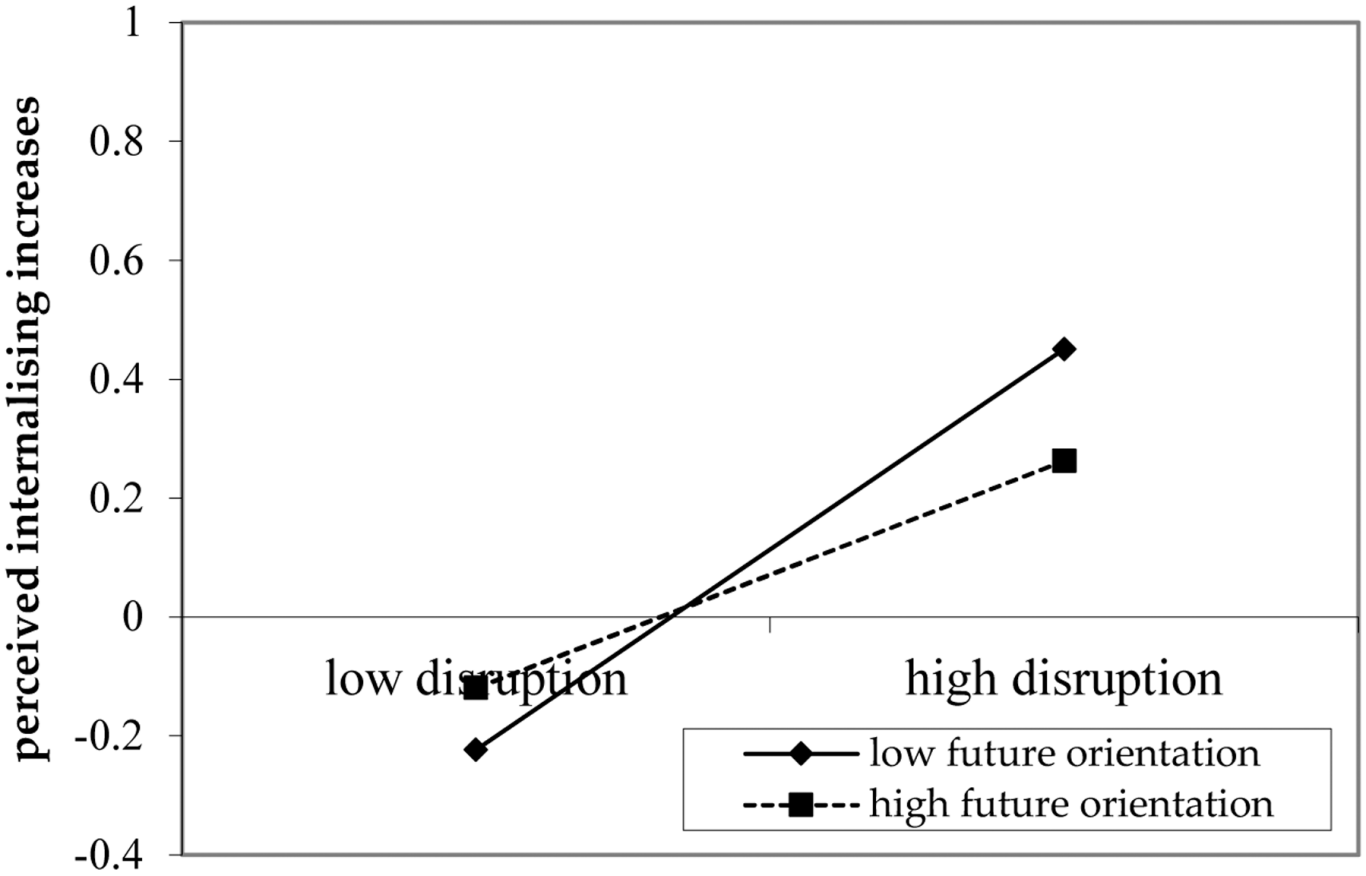
Moderation by Future Orientation.

Across all countries, adolescent future orientation during adolescence was not significantly related to perceived increases in internalising behaviours in young adults at age 20. However, there was consistent evidence across countries that the relation between experienced disruption due to COVID-19 and perceived increases in internalising behaviours in young adults was moderated by adolescents’ future orientation (see Figure 4). Greater personal disruption was associated with greater perceived increases in internalising behaviours among young adults who reported lower levels of future orientation (*b* = 0.07, *SE* = 0.03, *p* < 0.05). Use of more future-oriented thoughts and behaviours such as planning ahead during adolescence may thus have a protective role in the association between disruption due to COVID-19 and perceived increases in internalising behaviours in young adults. This association had a small effect size (Cohen 1992).

#### 8.1.4. Parental Psychological Control

**Figure 5.**
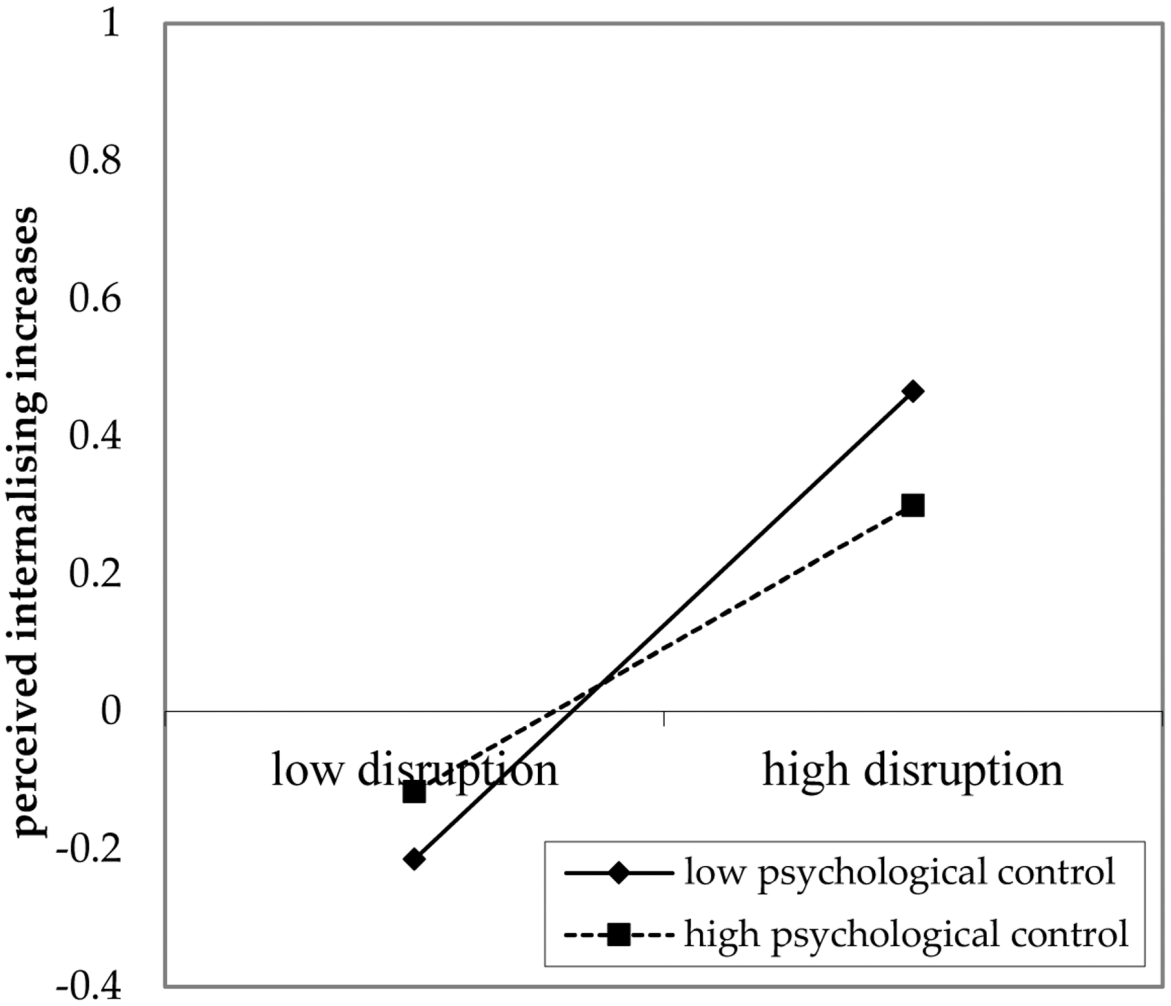
Moderation by Parental Psychological Control.

Across all countries, parental psychological control was not significantly related to perceived increases in internalising behaviours in young adults at age 20. However, across all countries, the relation between experienced disruption due to COVID-19 and perceived increases in internalising behaviours in young adults was moderated by parental psychological control during adolescence (see Figure 5). Greater personal disruption was associated with greater perceived increases in internalising behaviours among young adults who had lower levels of parental psychological control during adolescence (*b* = 0.07, *SE* = 0.03, *p* < 0.05). Higher levels of parental psychological control during adolescence may thus have a protective role in the association between disruption due to COVID-19 and perceived increases in internalising behaviours in young adults. This association also had a small effect size (Cohen 1992).

## 9. Discussion

This study examined the relation between COVID-19 personal disruption during the pandemic and perceived increases in young adults’ internalising symptoms, capitalising on a nine-country study in which pre-pandemic levels of internalising symptoms, positivity, future orientation, and parental psychological control during adolescence could be studied. Because the pandemic disrupted major developmental opportunities for growth, the young adult population is particularly compelling to study. In our sample, 52% of young adults reported their anxiety had increased as compared to before the pandemic, and 35% reported increases in depression, further highlighting the need to learn more about ways to dampen such impacts in future crises. This finding is in line with prior research during the COVID-19 pandemic showing the young adult population is especially vulnerable; although clinical levels of internalising symptoms were higher than national averages among young adults even before the pandemic began, significant increases were reported by young adults as early as 1–2 months after the pandemic began (Lee et al. 2020).

Our first hypothesis, that higher levels of self-reported disruption would be associated with perceived increases in internalising behaviour as compared to before the pandemic, was supported, with a 1 *SD* increase in the level of disruption associated with a 0.27 *SD* perceived increase in internalising symptoms. Increases in anxiety and depression among the young adult population, especially during times where access to leisure activities, social support, and medical and mental health services are reduced, are of concern because of their potential to impact future well-being, engagement in work and leisure activities, and physical health (Masten 2021; Masten and Motti-Stefanidi 2020).

With one exception in one country (Italy), adolescent positivity was associated with perceived increases in internalising behaviours during the pandemic, but no significant direct relations were found between adolescent future-orientation or parental psychological control and perceived increases in young adult internalising. Consistent with our hypotheses, positivity and future orientation during adolescence moderated the relation between pandemic disruption and perceived increases in internalising behaviour, suggesting a protective effect of future-oriented thinking and behaviour. McElroy et al. (2020) distinguished between two types of anxiety that arose due to the pandemic: disease anxiety and consequence anxiety. They found that, while some people (e.g., at-risk groups with physical health conditions) were worried about the disease itself, older adolescents and young adults were most concerned about the consequences of the pandemic. As such, this study shows that increases in internalising symptoms among young adults may be associated with pandemic-related factors related to long-term consequences for their future, such as educational and economic disruptions (Ahmed et al. 2020; McElroy et al. 2020).

Surprisingly, and contrary to our hypothesis, higher levels of psychological control during adolescence also served a buffering role between pandemic-related disruption and perceived increases in internalising behaviours. Although parental psychological control is consistently linked to higher levels of maladjustment for children, we speculate that the nature of the pandemic played a role in this unusual finding. The literature on community-wide stressors such as natural disasters and long-standing political violence provides some clues to why control may serve a protective role in its possible provisions of emotional security (see Cummings et al. 2010). During circumstances where multiple ecosystem levels are disrupted, and where physical and emotional health and well-being are threatened, young adults who might otherwise be disempowered by high levels of parental psychological control may instead—in these unusual circumstances—find predictability and consistency in a more controlling parent–child relationship.

The development of resiliency among young adults during the pandemic may come via several pathways. Parents can play a key role during childhood and adolescence to help buffer the negative impact of future community-wide stressors or other disruptive events in young adult lives. Through parent–child interaction, modelling interests and goals, and verbalising future-oriented thinking and optimism about personal goals and education (Kerpelman et al. 2008), parents set normative standards that can be adopted by adolescents in their thoughts and behaviours (Nurmi 1991). Some aspects of parental control may also serve a protective role during the pandemic. In one study with Dutch adolescents and their parents, the authors found that although youth reported an increase in rules set by parents during the pandemic and a temporary decrease in autonomy, most youth reported they felt these additional restrictions were legitimate and warranted (Bülow et al. 2021). Thus, if parents and young adults resided together during the pandemic and similar or even higher levels of control compared to before the pandemic were present, young adults may have understood these parenting behaviours to be protective. Further, given that the relation between psychological control and depressive symptoms is stronger for those with poor sadness regulation (Cui et al. 2014), it is possible the pandemic may have provided an opportunity for families to engage in more positive family interactions during the pandemic (see Bülow et al. 2021). If positive family interactions provided a way to increase emotion regulation skills among young adults, this could account for the diminished impact of psychological control on adjustment, which is typically maladaptive.

The literature on adverse childhood experiences (ACEs) also informs our understanding of other ways in which resilience may be developed as the impact of ACEs on biological, epigenetic, and psychosocial outcomes is realised (Anda et al. 2006; Merrick et al. 2017). The literature on ACEs suggests an ecological systems approach, as families and communities must be provided the skills, resources and support to overcome adversity (Rosanbalm et al. 2020). Empirically informed approaches to public policy and treatment practices (see, e.g., Blair et al. 2019) can improve outcomes for families and conserve limited resources.

### 9.1. Strengths and Limitations

The findings from this study should be interpreted in the context of several strengths and limitations to guide future research. Although this study included only self-report data, which is somewhat limiting in scope due to possible reporter bias, both pre-pandemic and pandemic data were utilised, and prior levels of internalising behaviours were controlled to rule out the possibility that increases in internalising behaviours were experienced only by those who were already experiencing symptoms of anxiety and depression before pandemic onset. Reporting about positivity is also best performed by adolescents themselves, as outside observers are not reliable reporters of thoughts and experiences of optimism (Caprara et al. 2019). Second, this study also included a measure not just of optimism and positive thinking (positivity), but of behaviours associated with planning, time perspective, and anticipation of future consequences (future orientation), which signals behavioural changes that can accompany positive thoughts and views about the future. Parents’ reports about their own positivity and future orientation or child reports about their perceptions of their parents’ behaviour would strengthen the argument that parents are able to influence their adolescents’ experiences with future-oriented thoughts and behaviour, especially during times of stress. Because our measure of disruption included a single item, we are only able to base our conclusions on how well an overall feeling of disruption predicts perceived changes in internalising behaviour and are not able to distinguish if these relations were due to disruptions in different areas, such as school, home, work, or social interaction. It is also possible that the time elapsed between community school closures and data collection may impact individual perceptions of disruption. For example, individuals who responded to the survey shortly into lockdowns may perceive disruption and increases in internalising to a lesser to degree than those who completed the survey after a few months of closures. However, we included a control variable of “weeks since the pandemic began” to minimise the possibility of spurious relations between the predictor and outcome. Because parental psychological control was measured during adolescence rather than concurrently with measures of pandemic disruption and perceived increases in internalising behaviours, we cannot be sure that psychological control has the same protective association were it to occur within the time period of the community-wide stressor. Indeed, psychological control typically has a negative impact on adjustment. Thus, a concurrent measure of all three moderators would inform our understanding not only of how parenting behaviours and youth perceptions change over time, but also whether the associations among the moderators we measured and the disruption–internalising link persist within a single time point during young adulthood. Finally, future work that pairs self-report measures of future orientation with observable behavioural measures of planning would add to the richness of the data.

### 9.2. Relevance for Parenting and Parent–Child Relationships in the 21st Century

Parents and children in the 21st century face a range of community-wide stressors with unclear solutions. Climate change, sectarian violence, and economic volatility, to name a few, promise to influence development around the world. Although uncertainty about the security of future events can increase parental stress and negatively impact parenting, exposure to some stressors can also contribute to resilience in children as parents try to restore a sense of “normality” (Prime et al. 2020) and emotional security. Resilience literature (see Masten 2015) emphasises several important applicable concepts. First, co-occurring stressors will disproportionately impact low-income countries and families. Thus, family and organisational supports are especially needed in already at-risk communities in order to shore up resources for additional community-wide stressors. Second, part of the distinction between maladjustment and resilience when facing stressors may lie in parents’ ability to engage youth in recovery planning, foster feelings of self-efficacy, and establish hope for the future (Masten and Motti-Stefanidi 2020). Recent research supports the idea that parental focus on future orientation can help buffer the impact of negative life events across a variety of settings. In a longitudinal study with adolescents in the United States, for example, parental involvement in activities of middle adolescence and parental advice about the future predicted adolescent positive adjustment and occupational goals two years later (Lee and Yu 2017).

Child development theories offer several explanations for how parenting may impact children’s future-oriented thinking and behaviour. For example, Seginer’s (2008) work with children exposed to political violence showed that children need exposure to challenges to develop resilience and that the development of hope is an important mediator in the link between challenge/resilience and future orientation (see also Zoellner and Maercker 2006). Further, research with youth exposed to community and political violence highlights that adolescents may be more vulnerable to psychological effects of stress exposure than younger children (Shaw 2003). Parenting is one resource for reducing adverse impacts on children’s social-emotional development (Perrin et al. 2016), and although the similarities between the COVID-19 pandemic and exposure to other community-wide stressors are yet to be confirmed, this study suggests that, if future orientation and positivity can be shaped by parents, parent–child relationships during adolescence could play a role in buffering the impact of life disruption into young adulthood. The resilience literature on ACEs (see Section 9, above) also supports the idea that efforts to improve outcomes for families facing threats to psychological and psychosocial health are best implemented from an empirically driven, community focused (e.g., schools, health service providers, and government) coordinated effort.

This study also provided new information about the role that parental psychological control may play in adjustment of young adults during a community-wide stressor. Though more research is needed to unpack which aspects and to what degree psychological control may serve a protective role during exposure to widespread threats to health and safety, the 21st century is ripe with opportunities for study. Researchers can use technology to gather more detailed information about daily fluctuations in the parent–child relationship and in youth responses to stressors, by using ecological momentary assessment or conducting studies of social media use.

## 10. Conclusions

This study demonstrated that across a wide range of sites in nine countries, with varying experiences and responses to the pandemic, adolescent positivity, future orientation, and parental psychological control during adolescence moderated the relation between disruption due to the COVID-19 pandemic and perceived increases in internalising symptoms several years later. Consistently across sites, we found that higher levels of adolescent positivity and future orientation may protect young adults from stronger relations between disruption and symptoms of depression and anxiety compared to young adults with comparatively lower adolescent levels of positivity and future orientation. Similarly, in contrast with prior research, we also found evidence that parental psychological control during adolescence may have provided some stability and predictability necessary to buffer the relation between pandemic disruption and young adults’ perceived increases in internalising symptoms. As the third and fourth decades of the 21st century may include continued disruptions to typical developmental trajectories, these findings inform our understanding of associations among long-studied psychological constructs during community-wide stressful experiences.

## Figures and Tables

**Table 1.** Sample Sizes (*n*), Means (*M*), Standard Deviations (*SD*), and Correlations of Main Study Variables.

Pearson Correlations												
Variables	*n*	*M*	*SD*	1	2	3	4	5	6	7	8	9
1. Child gender	1329	-	-	-								
2. Parents’ education	1318	13.71	4.18	0.00	-							
3. Weeks since pandemic began	810	20.41	12.1	0.00	−0.28 **	-						
4. Pandemic disruption	802	6.21	2.48	−0.13 **	0.05	−0.13 **	-					
5. Adolescent internalising	1042	14.1	8.97	−0.23 **	−0.02	−0.001	0.08 *	-				
6. Perceived internalising increases during pandemic	810	4.51	1.71	−0.27 **	0.10 **	−0.06	0.30 **	0.26 **	-			
7. Positivity	1039	3.92	0.65	0.01	0.03	−0.01	0.11 **	−0.47 **	−0.05	-		
8. Future orientation	998	2.98	0.51	−0.07 *	0.02	0.13 **	0.10 **	0.01	0.03	0.10 **	-	
9. Psychological control	1065	2.09	0.39	0.06	−0.34 **	0.14 **	0.09 *	0.00	−0.06	0.07 *	−0.03	-

* *p* < 0.05.

** *p* < 0.01.

**Table 2.** FIML Multiple Group Model Results.

	Main Model	Moderation by Positivity	Moderation by Future Orientation	Moderation by Parental Psycholological Control
Variables	Increases in Internalising Behaviours *b* (SE)			
Child gender	−0.48 (0.07) **	−0.50 (0.07) **	−0.44 (0.07) **	−0.49 (0.07) **
Parents’ education	0.07 (0.034) *	0.07 (0.03) *	0.07 (0.03) *	0.07 (0.03) **
Weeks since pandemic began	−0.01 (0.06)	−0.01 (0.06)	−0.01 (0.06)	−0.01 (0.06)
Pandemic disruption	0.27 (0.03) **	0.28 (0.03) **	0.26 (0.03) **	0.27 (0.03) **
Adolescent internalising	0.16 (0.04) **	0.15 (0.04) **	0.16 (0.04) **	0.16 (0.04) **
Positivity	0.12 (0.046) *	0.10 (0.47) *	0.12 (0.05) *	0.13 (0.05) *
Future orientation	0.01 (0.04)	−0.01 (0.03)	−0.02 (0.03)	−0.01 (0.03)
Psychological control	−0.02 (0.04)	0.00 (0.04)	−0.01 (0.04)	−0.02 (0.04)
Positivity × pandemic disruption	-	−0.09 (0.03) *	-	-
Future orientation × pandemic disruption	-	-	−0.07 (0.03) *	-
Psychological control × pandemic disruption	-	-	-	−0.07 (0.03) *
*Country-Specific Coefficients*				
Kenya—child gender	0.17 (0.20)	0.14 (0.20)	0.13 (0.20)	0.16 (0.20)
Thailand—child gender	−0.02 (0.18)	−0.03 (0.18)	-	−0.02 (0.18)
Italy—positivity	−0.17 (0.07) *	−0.19 (0.07) *	−0.17 (0.08) *	−0.17 (0.07) *
Kenya—positivity	0.12 (0.05) *	-	-	-
US—positivity	−0.12 (0.07)	−0.10 (0.07)	−0.12 (0.07)	−0.11 (0.07)
Colombia—future orientation	−0.25 (0.10) *	-	-	-
Jordan—weeks since pandemic began	0.72 (0.23) *	0.71 (0.23) *	0.72 (0.23) *	0.71 (0.23) *
*Model Fit Statistics*				
Chi-square test (degrees of freedom), *p*-value	66.59 (58), 0.2	75.71 (67), 0.22	79.27 (68), 0.16	78.48 (67), 0.15
CFI/TLI	0.96/0.95	0.96/0.95	0.96/0.94	0.95/0.93
RMSEA	0.03	0.03	0.03	0.03
SRMR	0.03	0.03	0.03	0.03

*Note*. B = Standardised coefficient. CFI = the comparative fit index; TLI = the Tucker–Lewis index; RMSEA = the root mean square error of approximation; SRMR = standardised root mean square residual.

* *p* < 0.05.

** *p* < 0.01.

## Data Availability

The data presented in this study are available on request from the corresponding author. The data are not publicly available due to privacy and ethics restrictions.

## References

[R1] Achenbach Thomas M. 1991. Integrative Guide for the 1991 CBCL/4–18, YSR, and TRF Profiles. Burlington: Department of Psychiatry, University of Vermont.

[R2] Ahmed Faheem, Ahmed Na’eem, Pissarides Christopher, and Stiglitz Joseph. 2020. Why inequality could spread COVID-19. The Lancet Public Health 5: e240. 32247329 10.1016/S2468-2667(20)30085-2 PMC7270465

[R3] Jazeera Al. 2021. Kenya Bans In-Person Meetings, Public Gatherings as COVID Surges. Available online: https://www.aljazeera.com/news/2021/7/30/kenya-bans-in-person-meetings-public-gatherings-as-covid-surges (accessed on 26 October 2021).

[R4] Alessandri Guido, Zuffianò Antonio, Fabes Richard, Vecchione Michele, and Martin Carol. 2014. Linking positive affect and positive self-beliefs in daily life. Journal of Happiness Studies 15: 1479–93.

[R5] Alessandri Guido, Caprara Gian Vittorio, and Tisak John. 2012. The unique contribution of positive orientation to optimal functioning. European Psychologist 17: 44–54.

[R6] Anda Robert F., Felitti Vincent J., Bremner J. Douglas, Walker John D., Whitfield CH, Perry Bruce D., Dube Sh R., and Giles Wayne H.. 2006. The enduring effects of abuse and related adverse experiences in childhood. European Archives of Psychiatry and Clinical Neuroscience 256: 174–86. 16311898 10.1007/s00406-005-0624-4 PMC3232061

[R7] Andersen Astrid Juhl, Mary-Krause Murielle, Herranz Bustamante Joel José, Héron Mégane, Aarbaoui Tarik El, and Melchior Maria. 2021. Symptoms of anxiety/depression during the COVID-19 pandemic and associated lockdown in the community: Longitudinal data from the TEMPO cohort in France. BMC Psychiatry 21: 381. 34320943 10.1186/s12888-021-03383-z PMC8316881

[R8] Arredondo Elva M., Elder John P., Ayala Guadalupe X., Campbell Nadia, Baquero Barbara, and Duerksen Susan. 2006. Is parenting style related to children’s healthy eating and physical activity in Latino families? Health Education Research 21: 862–71. 17032706 10.1093/her/cyl110

[R9] Arslan Gökmen, and Yıldırım Murat. 2021. Coronavirus stress, meaningful living, optimism, and depressive symptoms: A study of moderated mediation model. Australian Journal of Psychology 73: 113–24.

[R10] Barber Brian K. 1996. Parental psychological control: Revisiting a neglected construct. Child Development 67: 3296–319. 9071782

[R11] Barber Brian K., and Xia Mingzhu. 2013. The centrality of control to parenting and its effects. In Authoritative Parenting: Synthesizing Nurturance and Discipline for Optimal Child Development. Washington, DC: American Psychological Association, pp. 61–87.

[R12] Barber Brian K., Olsen Joseph E., and Shagle Shobha C.. 1994. Association between parental psychological and behavioral control and youth internalized and externatilzed behavior. Child Development 65: 1120–36. 7956469

[R13] Bermudez Laura Gauer, Stark Lindsay, Bennouna Cyril, Jensen Celina, Potts Alina, Kaloga Inah Fatoumata, Tilus Ricardo, Buteauf Jean Emmanuel, Marshg Mendy, Hooverc Anna, and 2019. Converging drivers of interpersonal violence: Findings from a qualitative study in post-hurricane Haiti. Child Abuse & Neglect 89: 178–91. 30685625 10.1016/j.chiabu.2019.01.003

[R14] Best R 2021. Coronavirus (COVID-19) Deaths Worldwide per One Million Population as of August 17, 2021, by Country [Data Set]. Statista. Available online: https://www.statista.com/statistics/1104709/coronavirus-deaths-worldwide-per-million-inhabitants/ (accessed on 17 August 2021).

[R15] Blair Katelyn, Topitzes James, and Mersky Joshua P.. 2019. Do parents’ adverse childhood experiences influence treatment responses to parent-child interaction therapy? An exploratory study with a child welfare sample. Child & Family Behavior Therapy 41: 73–83.

[R16] Brown Timothy A. 2006. Confirmatory Factor Analysis for Applied Research. New York: Guilford Press.

[R17] Bubola E 2021. Italy says it will require proof of vaccination or a negative test for many social activities. The New York Times. Available online: https://www.nytimes.com/2021/07/22/world/europe/italy-covid-vaccine-proof-activities.html (accessed on 26 October 2021).

[R18] Bülow Anne, Keijsers Loes, Boele Savannah, van Roekel Eeske, and Denissen Jaap J. A.. 2021. Parenting adolescents in times of a pandemic: Changes in relationship quality, autonomy support, and parental control? Developmental Psychology 57: 1582. 34807682 10.1037/dev0001208

[R19] Calderoni Michele E., Alderman Elizabeth M., Silver Ellen J., and Bauman Laurie J.. 2006. The mental health impact of 9/11 on inner-city high school students 20 miles north of Ground Zero. Journal of Adolescent Health 39: 57–65. 10.1016/j.jadohealth.2005.08.012 16781962

[R20] Caprara Gian Vittorio, Alessandri Guido, and Caprara Mariagiovanna. 2019. Associations of positive orientation with health and psychosocial adaptation: A review of findings and perspectives. Asian Journal of Social Psychology 22: 126–32.

[R21] Caprara Gian Vittorio, Alessandri Guido, Eisenberg Nancy, Kupfer A, Steca Patrizia, Caprara Maria Giovanna, Yamaguchi Susumu, Fukuzawa Ai, and Abela John. 2012. The positivity scale. Psychological Assessment 24: 701–12. 22250591 10.1037/a0026681

[R22] Caprara Mariagiovanna, Di Giunta Laura, and Caprara Gian Vittorio. 2017. Association of positivity with health problems in old age: Preliminary findings from Spanish middle class seniors. Journal of Happiness Studies 18: 1339–58.

[R23] Chao Ruth K., and Aque Christine. 2009. Interpretations of parental control by Asian immigrant and European American youth. Journal of Family Psychology 23: 342. 19586197 10.1037/a0015828

[R24] Cohen Jacob. 1992. A power primer. Psychological Bulletin 112: 155–59. 19565683 10.1037//0033-2909.112.1.155

[R25] Cui Lixian, Morris Amanda Sheffield, Criss Michael M., Houltberg Benjamin J., and Silk Jennifer S.. 2014. Parental psychological control and adolescent adjustment: The role of adolescent emotion regulation. Parenting 14: 47–67. 25057264 10.1080/15295192.2014.880018 PMC4104177

[R26] Cui Ming, and Hong Peipei. 2021. COVID-19 and mental health of young adult children in China: Economic impact, family dynamics, and resilience. Family Relations 70: 1358–68. 34548727 10.1111/fare.12573 PMC8444862

[R27] Cummings E. Mark, Merrilees Christine E., Schermerhorn Alice C., Goeke-Morey Marcie C., Shirlow Peter, and Cairns Ed. 2010. Testing a social ecological model for relations between political violence and child adjustment in Northern Ireland. Development and Psychopathology 22: 405–18. 20423550 10.1017/S0954579410000143 PMC3712527

[R28] Davidson Bridget, Schmidt Ellyn, Mallar Carolina, Mahmoud Farah, Rothenberg William, Hernandez Julieta, Berkovits Michelle, Jent Jason, Delamater Alan, and Natale Ruby. 2020. Risk and resilience of well-being in caregivers of young children in response to the COVID-19 pandemic. Translational Behavioral Medicine 11: 305–13. 10.1093/tbm/ibaa124 PMC7890655 33236766

[R29] Dornbusch Sanford M., Carlsmith J. Merrill, Bushwall Steven J., Ritter Philip L., Leiderman Herbert, Hastorf Albert H., and Gross Ruth T.. 1985. Single parents, extended households, and the control of adolescents. Child Development 56: 326–41. 3987411

[R30] Erkut Sumru. 2010. Developing multiple language versions of instruments for intercultural research. Child Development Perspectives 4: 19–24. 21423824 10.1111/j.1750-8606.2009.00111.x PMC3060794

[R31] Faherty Amanda N., Lowe Katie, and Arnett Jeffrey Jensen. 2020. Mind games: Parental psychological control and emerging adults’ adjustment. Journal of Social and Personal Relationships 37: 695–714.

[R32] Fegert Jörg M., Vitiello Benedetto, Plener Paul L., and Clemens Vera. 2020. Challenges and burden of the Coronavirus 2019 (COVID-19) pandemic for child and adolescent mental health: A narrative review to highlight clinical and research needs in the acute phase and the long return to normality. Child and Adolescent Psychiatry and Mental Health 14: 1–11. 32419840 10.1186/s13034-020-00329-3 PMC7216870

[R33] Fredrickson Barbara L., Tugade Michele M., Waugh Christian E., and Larkin Gregory R.. 2003. What good are positive emotions in crisis? A prospective study of resilience and emotions following the terrorist attacks on the United States on September 11th, 2001. Journal of Personality and Social Psychology 84: 365–76. 12585810 10.1037//0022-3514.84.2.365 PMC2755263

[R34] Frigerio Alessandra, Rucci Paola, Goodman Robert, Ammaniti Massimo, Carlet Ombretta, Cavolina Pina, De Girolamo Giovanni, Lenti Carlo, Lucarelli Loredana, Mani Elisa, and 2009. Prevalence and correlates of mental disorders among adolescents in Italy: The PrISMA study. European Child & Adolescent Psychiatry 18: 217–26. 19165539 10.1007/s00787-008-0720-x

[R35] Fulton Colm. 2021. COVID-19 on the Rise in Swedish Cities as Delta Outbreaks Dominate. London: Reuters. Available online: https://www.reuters.com/world/europe/covid-19-rise-swedish-cities-delta-outbreaks-dominate-2021-07-23/ (accessed on 26 October 2021).

[R36] Gan Nectar. 2021. China Issues New Guidelines for Face Mask Wearing Amid Delta Outbreak. Atlanta: CNN. Available online: https://edition.cnn.com/2021/08/13/china/china-covid-mask-guideline-intl-hnk/index.html (accessed on 26 October 2021).

[R37] Goodman Peter S. 2020. Sweden has become the world’s cautionary tale. The New York Times. Available online: https://www.nytimes.com/2020/07/07/business/sweden-economy-coronavirus.html (accessed on 26 October 2021).

[R38] Gray Marjory Roberts, and Steinberg Laurence. 1999. Unpacking authoritative parenting: Reassessing a multidimensional construct. Journal of Marriage and the Family 61: 574–87.

[R39] Gritti Antonella, Bravaccio Carmela, Signoriello Simona, Salerno Filomena, Pisano Simone, Catone Gennaro, Gallo Ciro, and Pascotto Antonio. 2014. Epidemiological study on behavioural and emotional problems in developmental age: Prevalence in a sample of Italian children, based on parent and teacher reports. Italian Journal of Pediatrics 40: 1–7. 24533835 10.1186/1824-7288-40-19 PMC3972618

[R40] Hafstad Gertrud S., Haavind Hanne, and Jensen Tine K.. 2012. Parenting after a natural disaster: A qualitative study of Norwegian families surviving the 2004 tsunami in Southeast Asia. Journal of Child and Family Studies 21: 293–302. 22448107 10.1007/s10826-011-9474-z PMC3304071

[R41] Hamilton Jessica L., Connolly Samantha L., Liu Richard T., Stange Jonathan P., Abramson Lyn Y., and Alloy Lauren B.. 2015. It gets better: Future orientation buffers the development of hopelessness and depressive symptoms following emotional victimization during early adolescence. Journal of Abnormal Child Psychology 43: 465–74. 25052625 10.1007/s10802-014-9913-6 PMC4305347

[R42] Harter Susan. 1982. The perceived competence scale for children. Child Development 53: 87–97. 6525886

[R43] Hawryluck Laura, Gold Wayne L., Robinson Susan, Pogorski Stephen, Galea Sandro, and Styra Rima. 2004. SARS control and psychological effects of quarantine, Toronto, Canada. Emerging Infectious Diseases 10: 1206. 15324539 10.3201/eid1007.030703 PMC3323345

[R44] Hendricks Charlene, and Bornstein Marc H.. 2007. Ecological analysis of early adolescents’ stress responses to 9/11 in the Washington, DC, area. Applied Development Science 11: 71–88.

[R45] Holman E. Alison, and Silver Roxane Cohen. 2005. Future-oriented thinking and adjustment in a nationwide longitudinal study following the September 11th terrorist attacks. Motivation and Emotion 29: 385–406.

[R46] Islam Md Saiful, Tasnim Rafia, Sujan Md Safaet Hossain, Ferdous Most Zannatul, Sikder Md Tajuddin, Masud Jakir Hossain Bhuiyan, Kundu Sourav, Tahsin Promi, Mosaddek Abu Syed Md, and Griffiths Mark D.. 2021. Depressive symptoms associated with COVID-19 preventive practice measures, daily activities in home quarantine and suicidal behaviors: Findings from a large-scale online survey in Bangladesh. BMC Psychiatry 21: 273. 34039292 10.1186/s12888-021-03246-7 PMC8150150

[R47] Johnson Sarah R. Lindstrom, Blum Robert W., and Cheng Tina L.. 2014. Future orientation: A construct with implications for adolescent health and wellbeing. International Journal of Adolescent Medicine and Health 26: 459–68. 24523304 10.1515/ijamh-2013-0333 PMC4827712

[R48] Kapetanovic Sabina, Gurdal Sevtap, Ander Birgitta, and Sorbring Emma. 2021. Reported changes in adolescent psychosocial functioning during the COVID-19 outbreak. Adolescents 1: 10–20.

[R49] Kaplan Carole A., and Owens Julie. 2004. Parental influences on vulnerability and resilience. In Handbook of Parenting: Theory and Research for Practice. London: SAGE Publications Ltd., pp. 72–87.

[R50] Kecojevic Aleksandar, Basch Corey H., Sullivan Marianne, and Davi Nicole K.. 2020. The impact of the COVID-19 epidemic on mental health of undergraduate students in New Jersey, cross-sectional study. PLoS ONE 15: e0239696. 32997683 10.1371/journal.pone.0239696 PMC7526896

[R51] Kelloway E. Kevin. 2014. Using Mplus for Structural Equation Modeling: A Researcher’s Guide. Thousand Oaks: Sage Publications.

[R52] Kerpelman Jennifer L., Eryigit Suna, and Stephens Carolyn J.. 2008. African American adolescents’ future education orientation: Associations with self-efficacy, ethnic identity, and perceived parental support. Journal of Youth and Adolescence 37: 997–1008.

[R53] Kline Rex B. 2005. Principles and Practice of Structural Equation Modeling, 2nd ed. New York: Guilford Press.

[R54] Lalot Fanny, Abrams Dominic, Ahvenharju Sanna, and Minkkinen Matti. 2021. Being future-conscious during a global crisis: The protective effect of heightened Futures Consciousness in the COVID-19 pandemic. Personality and Individual Differences 178: 110862. 36540789 10.1016/j.paid.2021.110862 PMC9755894

[R55] Lansford Jennifer E., Bornstein Marc H., Deater-Deckard Kirby, Dodge Kenneth A., Al-Hassan Suha M., Bacchini Dario, Bombi Anna Silvia, Chang Lei, Chen Bin-Bin, Di Giunta Laura, and 2016. How international research on parenting advances understanding of child development. Child Development Perspectives 10: 202–7. 27725843 10.1111/cdep.12186 PMC5054977

[R56] Lee Christine M., Cadigan Jennifer M., and Rhew Isaac C.. 2020. Increases in loneliness among young adults during the COVID-19 pandemic and association with increases in mental health problems. Journal of Adolescent Health 67: 714–17. 10.1016/j.jadohealth.2020.08.009 PMC7576375 33099414

[R57] Lee Sun-A., and Yu Jeong Jin. 2017. Parenting, adolescents’ future orientation, and adolescents’ efficient financial behaviors in young adulthood. Journal of Social Sciences 13: 197–207.

[R58] Lopez-Carr A 2021. Covid-19 Cases Rise as Delta Variant Sweeps the Globe. Santa Barbara: DirectRelief. Available online: https://www.directrelief.org/2021/08/covid-cases-rise-as-delta-variant-sweeps-the-globe/ (accessed on 26 October 2021).

[R59] Ma Xiaole, and Wang Xingchao. 2021. The role of empathy in the mechanism linking parental psychological control to emotional reactivities to COVID-19 pandemic: A pilot study among Chinese emerging adults. Personality and Individual Differences 168: 110399. 32982001 10.1016/j.paid.2020.110399 PMC7500908

[R60] MacLeod Andrew K., and Conway Clare. 2005. Well-being and the anticipation of future positive experiences: The role of income, social networks, and planning ability. Cognition & Emotion 19: 357–74. 22686648 10.1080/02699930441000247

[R61] Mandara Jelani, and Pikes Crysta L.. 2008. Guilt trips and love withdrawal: Does mothers’ use of psychological control predict depressive symptoms among African American adolescents? Family Relations 57: 602–12.

[R62] Manzeske David P., and Stright Anne Dopkins. 2009. Parenting styles and emotion regulation: The role of behavioral and psychological control during young adulthood. Journal of Adult Development 16: 223.

[R63] Masten Ann S. 2015. Ordinary Magic: Resilience in Development. New York: Guilford Publications.

[R64] Masten Ann S. 2021. Resilience of children in disasters: A multisystem perspective. International Journal of Psychology 56: 1–11. 33325580 10.1002/ijop.12737

[R65] Masten Ann S., and Motti-Stefanidi Frosso. 2020. Multisystem resilience for children and youth in disaster: Reflections in the context of COVID-19. Adversity and Resilience Science 1: 95–106. 32838305 10.1007/s42844-020-00010-w PMC7314620

[R66] McCann Allison, Popovich Nadja, and Wu Jin. 2020. Italy’s Virus Shutdown Came Too late. What Happens Now? Available online: https://www.nytimes.com/interactive/2020/04/05/world/europe/italy-coronavirus-lockdown-reopen.html (accessed on 26 October 2021).

[R67] McElroy Eoin, Patalay Praveetha, Moltrecht Bettina, Shevlin Mark, Shum Adrienne, Creswell Cathy, and Waite Polly. 2020. Demographic and health factors associated with pandemic anxiety in the context of COVID-19. British Journal of Health Psychology 25: 934–44. 32860334 10.1111/bjhp.12470

[R68] Merrick Melissa T., Ports Katie A., Ford Derek C., Afifi Tracie O., Gershoff Elizabeth T., and Grogan-Kaylor Andrew. 2017. Unpacking the impact of adverse childhood experiences on adult mental health. Child Abuse & Neglect 69: 10–19. 28419887 10.1016/j.chiabu.2017.03.016 PMC6007802

[R69] Milioni Michela, Alessandri Guido, Eisenberg Nancy, and Caprara Gian Vittorio. 2016. The role of positivity as predictor of ego-resiliency from adolescence to young adulthood. Personality and Individual Differences 101: 306–11.

[R70] Mohiddin A, Temmerman M, and Adam R. 2020. Kenya: What Kenya Needs to Do Better As It Braces for Fourth Wave of COVID-19. Available online: https://allafrica.com/stories/202108020022.html (accessed on 26 October 2021).

[R71] Mönks F 1968. Future time perspective in adolescents. Human Development, 107–23. 10.1159/000270600 6078970

[R72] Muthén LK, and Muthén BO. 2017. MPlus Version 8 [Computer Software]. Los Angeles: Muthén & Muthén.

[R73] Nurmi JE 1991. How do adolescents see their future? A review of the development of future orientation and planning. Developmental Review 11: 1–59.

[R74] Oettingen Gabriele, Mayer Doris, and Portnow Sam. 2016. Pleasure now, pain later: Positive fantasies about the future predict symptoms of depression. Psychological Science 27: 345–53. 26825106 10.1177/0956797615620783

[R75] Patterson Gerald R., and Stouthamer-Loeber Magda. 1984. The correlation of family management practices and delinquency. Child Development 55: 1299–307. 6488958

[R76] Perrin Ellen C., Leslie Laurel K., and Boat Thomas. 2016. Parenting as primary prevention. JAMA Pediatrics 170: 637–38. 27182902 10.1001/jamapediatrics.2016.0225

[R77] Pisano Simone, Catone Gennaro, Gritti Antonella, Almerico Luisa, Pezzella Anna, Santangelo Pia, Bravaccio Carmela, Iuliano Raffaella, and Senese Vincenzo Paolo. 2021. Emotional symptoms and their related factors in adolescents during the acute phase of Covid-19 outbreak in South Italy. Italian Journal of Pediatrics 47: 1–8. 33827644 10.1186/s13052-021-01036-1 PMC8026329

[R78] Prime Heather, Wade Mark, and Browne Dillon T.. 2020. Risk and resilience in family well-being during the COVID-19 pandemic. American Psychologist 75: 631–43. 32437181 10.1037/amp0000660

[R79] Racine Nicole, McArthur Brae Anne, Cooke Jessica E., Eirich Rachel, Zhu Jenney, and Madigan Sheri. 2021. Global prevalence of depressive and anxiety symptoms in children and adolescents during COVID-19: A meta-analysis. JAMA Pediatrics 175: 1142–50. 34369987 10.1001/jamapediatrics.2021.2482 PMC8353576

[R80] Ravens-Sieberer Ulrike, Kaman Anne, Otto Christiane, Adedeji Adekunle, Devine Janine, Erhart Michael, Napp Ann-Kathrin, Becker Marcia, Blanck-Stellmacher Ulrike, Löffler Constanze, and 2020. Mental health and quality of life in children and adolescents during the COVID-19 pandemic—Results of the COPSY study. Deutsches Ärzteblatt International 117: 828–29. 33568260 10.3238/arztebl.2020.0828 PMC8005842

[R81] Regan Helen. 2021. Delta Variant Is Ravaging the World but It’s Pushing Southeast Asia to Breaking Point. Hong Kong: CNN. Available online: https://www.cnn.com/2021/08/04/asia/southeast-asia-delta-covid-explainer-intl-hnk/index.html (accessed on 26 October 2021).

[R82] Reuters. 2021. Chart of Jordan’s COVID-19 Lockdown Implementations. Available online: https://graphics.reuters.com/world-coronavirus-tracker-and-maps/countries-and-territories/jordan/ (accessed on 15 August 2021).

[R83] Rodgers Robert R. 1966. Cornell Parent Behavior Description—An Interim Report. Ithaca: Department of Human Development and Family Studies, Cornell University.

[R84] Rogers Kelly N., Buchanan Christy M., and Winchell Megan E.. 2003. Psychological control during early adolescence: Links to adjustment in differing parent/adolescent dyads. The Journal of Early Adolescence 23: 349–83.

[R85] Rosanbalm Katie, DeKonty Elizabeth, and Fleming Sheronda. 2020. The North Carolina Resilience and Learning Project. In Alleviating the Educational Impact of Adverse Childhood Experiences. Charlotte: School-University-Community Collaboration, pp. 1–37.

[R86] Rueda M 2021. Colombia Loosens COVID Restrictions to Save the Economy as Deaths Soar. The World. Available online: https://www.pri.org/stories/2021-06-25/colombia-loosens-covid-restrictions-save-economy-deaths-soar (accessed on 26 October 2021).

[R87] Saddik Basema, Hussein Amal, Albanna Ammar, Elbarazi Iffat, Al-Shujairi Arwa, Temsah Mohamad-Hani, Sharif-Askari Fatemeh Saheb, Stip Emmanuel, Hamid Qutayba, and Halwani Rabih. 2021. The psychological impact of the COVID-19 pandemic on adults and children in the United Arab Emirates: A nationwide cross-sectional study. BMC Psychiatry 21: 224. 33941119 10.1186/s12888-021-03213-2 PMC8090921

[R88] Santucci Emily. 2020. What Lies Ahead as Jordan Faces the Fallout of COVID-19. Washington, DC: Atlantic Council. Available online: https://www.atlanticcouncil.org/blogs/menasource/what-lies-ahead-as-jordan-faces-the-fallout-of-covid-19/ (accessed on 26 October 2021).

[R89] Schafer Joseph L., and Graham John W.. 2002. Missing data: Our view of the state of the art. Psychological Methods 7: 147. 12090408

[R90] Seginer Rachel. 1986. Jewish-Arab relations in Israel: A psychology of adolescence perspective. The Journal of psychology 120: 557–65. 3820130 10.1080/00223980.1986.9915486

[R91] Seginer Rachel. 2009. The effect of parenting on future orientation. In Future Orientation: Developmental and Ecological Perspectives. Boston: Springer, pp. 125–47.

[R92] Seginer Rachel. 2008. Future orientation in times of threat and challenge: How resilient adolescents construct their future. International Journal of Behavioral Development 32: 272–82.

[R93] Shaw Jon A. 2003. Children exposed to war/terrorism. Clinical Child and Family Psychology Review 6: 237–46. 14719636 10.1023/b:ccfp.0000006291.10180.bd

[R94] Shing Elaine Z., Jayawickreme Eranda, and Waugh Christian E.. 2016. Contextual positive coping as a factor contributing to resilience after disasters. Journal of Clinical Psychology 72: 1287–306. 27410521 10.1002/jclp.22327

[R95] Sholomskas Diane, and Axelrod Rosalind. 1986. The influence of mother-daughter relationships on women’s sense of self and current role choices. Psychology of Women Quarterly 10: 171–82.

[R96] Silk Jennifer S., Morris Amanda S., Kanaya Tomoe, and Steinberg Laurence. 2003. Psychological control and autonomy granting: Opposite ends of a continuum or distinct constructs? Journal of Research on Adolescence 13: 113–28.

[R97] Skinner Ann T., Godwin Jennifer, Alampay Liane Peña, Lansford Jennifer E., Bacchini Dario, Bornstein Marc H., Deater-Deckard Kirby, Di Giunta Laura, Dodge Kenneth A., Gurdal Sevtap, and 2021. Parent-adolescent relationship quality as a moderator of links between COVID-19 disruption and reported changes in mothers and young adults’ adjustment in five countries. Developmental Psychology 57: 1648. 34807687 10.1037/dev0001236 PMC9590658

[R98] Smetana Judith G. 2017. Current research on parenting styles, dimensions, and beliefs. Current Opinion in Psychology 15: 19–25. 28813261 10.1016/j.copsyc.2017.02.012

[R99] Smith Lee, Jacob Louis, Yakkundi Anita, McDermott Daragh, Armstrong Nicola C., Barnett Yvonne, López-Sánchez Guillermo F., Martin Suzanne, Butler Laurie, and Tully Mark A.. 2020. Correlates of symptoms of anxiety and depression and mental wellbeing associated with COVID-19: A cross-sectional study of UK-based respondents. Psychiatry Research 291: 113138. 32562931 10.1016/j.psychres.2020.113138 PMC7258801

[R100] Smokowski Paul R., Guo Shenyang, Evans Caroline B. R., Wu Qi, Rose Roderick A., Bacallao Martica, and Cotter Katie L.. 2017. Risk and protective factors across multiple microsystems associated with internalizing symptoms and aggressive behavior in rural adolescents: Modeling longitudinal trajectories from the Rural Adaptation Project. American Journal of Orthopsychiatry 87: 94. 26881984 10.1037/ort0000163

[R101] So Suzanna, Gaylord-Harden Noni K., Voisin Dexter R., and Scott Darrick. 2018. Future orientation as a protective factor for African American adolescents exposed to community violence. Youth & Society 50: 734–57.

[R102] Soenens Bart, and Vansteenkiste Maarten. 2010. A theoretical upgrade of the concept of parental psychological control: Proposing new insights on the basis of self-determination theory. Developmental Review 30: 74–99.

[R103] Spencer Terry, and Kennedy Kelli. 2021. US Averaging 100,000 New COVID-19 Infections a Day. AP News. Available online: https://apnews.com/article/health-coronavirus-pandemic-b0811d7287ef240ae619ba1e385e0a63 (accessed on 26 October 2021).

[R104] Steinberg Laurence, SMounts Nina, Lamborn Susie D., and Dornbusch Sanford M.. 1991. Authoritative parenting and adolescent adjustment across various ecological niches. Journal of Research on Adolescence 1: 19–36.

[R105] Steinberg Laurence, Graham Sandra, O’brien Lia, Woolard Jennifer, Cauffman Elizabeth, and Banich Marie. 2009. Age differences in future orientation and delay discounting. Child Development 80: 28–44. 19236391 10.1111/j.1467-8624.2008.01244.x

[R106] Stoddard Sarah A., Zimmerman Marc A., and Bauermeister José A.. 2011. Thinking about the future as a way to succeed in the present: A longitudinal study of future orientation and violent behaviors among African American youth. American Journal of Community Psychology 48: 238–46. 21104432 10.1007/s10464-010-9383-0 PMC3107351

[R107] Weeland Joyce, Keijsers Loes, and Branje Susan. 2021. Introduction to the special issue: Parenting and family dynamics in times of the COVID-19 pandemic. Developmental Psychology 57: 1559. 34807680 10.1037/dev0001252

[R108] World Health Organization. 2004. Promoting Mental Health: Concepts, Emerging Evidence, Practice. Geneva: World Health Organization.

[R109] Zheng Lei, Lippke Sonia, Chen Yidi, Li Danyang, and Gan Yiqun. 2019. Future orientation buffers depression in daily and specific stress. PsyChi Journal 8: 342–52. 10.1002/pchj.283 30945435

[R110] Zimmerman Mark, Sheeran Thomas, and Young Diane. 2004. The Diagnostic Inventory for Depression: A self-report scale to diagnose DSM–IV major depressive disorder. Journal of Clinical Psychology 60: 87–110. 14692011 10.1002/jclp.10207

[R111] Zoellner Tanja, and Maercker Andreas. 2006. Posttraumatic growth in clinical psychology—A critical review and introduction of a two component model. Clinical Psychology Review 26: 626–53. 16515831 10.1016/j.cpr.2006.01.008

